# Bovine Congenital Defects Recorded in a National Survey of Dairy and Beef Herds Over Ten Years (2014–2023)

**DOI:** 10.1111/rda.70067

**Published:** 2025-04-30

**Authors:** Katie Quigley, John F. Mee

**Affiliations:** ^1^ Irish Cattle Breeding Federation Cork Ireland; ^2^ Animal & Bioscience Research Department Teagasc Cork Ireland

**Keywords:** congenital defects, intestinal atresia, multiple defects, national survey, tail abnormalities

## Abstract

This study describes a novel model of farm‐based congenital defect recording, with use of accessory data from a national breeding organisation. The study ran from 2014 to 2023 inclusive resulting in 522 reports of bovine congenital defects with additional data available for a subset (369/522). The most commonly reported defect was intestinal atresia (24.3%), followed by multiple defects (19.9%), and tail abnormalities (13.4%). This is the first national survey of bovine congenital defects in Ireland. It shows that while farmer‐reported cases generally lack a veterinary clinical or necropsy examination, farmers are in the unique position of being able to report phenotypic abnormalities first‐hand, and provide an unbiased perspective of the true typology and epidemiology of congenital defects on farm.

## 1 Introduction

Congenital abnormalities are those that are present at birth. These may be structural and/or functional anomalies and may affect one or more organ systems (Sağlam et al. 2022). In cattle, it is estimated that 0.5%–1% of calves are affected by congenital defects (Hobbenaghi et al. 2015) however, such estimates probably grossly underestimate true prevalence due to inaccurate recording.

Underlying causes may be genetic, non‐genetic, an interaction between both of those factors, or in many cases, unknown (Mee et al. 2024; Schalles et al. 2014). Non‐genetic factors describe those acquired by the dam during gestation; for example, ingestion of toxins or an acquired infectious disease (Agerholm et al. 2015). Genetic factors describe genetic variations that may be (1) heritable, that is, occur as a result of the anomaly being transmitted from one generation to the next or (2) non‐hereditary, that is, occur due to a spontaneous genetic mutation present in the foetus's genetic makeup but not present in the sire or dam, also known as a de novo mutation (Azevedo et al. 2024).

Genomic selection and reproductive technologies have accelerated advances in genetic gain in the cattle breeding industry, but have resulted in the widespread use of a smaller pool of high genetic merit sire lines compared to the pre genomic era. This decrease in effective population size gives rise to the increased frequency of deleterious alleles. This can be attributed to a reduction in genetic diversity and inbreeding due to the intensive selection of certain sire lines favouring the prevalence of homozygous regions within the genome (Doublet et al. 2019; Scott et al. 2021). Well known examples include the Holstein haplotypes affecting fertility and embryonic loss (Van Raden et al. 2011). Heritable conditions while rare (Derks and Steensma 2021) are often recessive in nature and so may not manifest for multiple generations (Sieck et al. 2020) However, the risk of even a dominant deleterious phenotype being spread in a population may be accelerated due to the widespread use of young genomically selected males. De novo mutations emerging each generation are an essential source of genetic diversity but also deleterious effects (Azevedo et al. 2024). The rate of de novo mutations, including those with deleterious impact arising during the early development of the embryo, may be accelerated with assisted reproductive technologies (Harland et al. 2018). Currently, the electronic database, Online Mendelian Inheritance in Animals (OMIA) lists 248 known likely causal variants for Mendelian diseases in cattle, spanning a multitude of phenotypes and breeds including both inherited and de novo variants (Nicholas et al. 2024).

Genomic data can be leveraged to aid the detection and management of genomic conditions amongst many other applications. The National Genotyping Program (NGP) in Ireland is a collaborative initiative set up in 2023 aiming to be the first country to eventually genotype the national cattle herd (DAFM 2023). The national cattle herd is composed of 44.3 k beef herds and 15.4 k dairy herds. Approximately 11% and 13% are pedigree herds, respectively. The average number of animals in a dairy herd is 145 and in a beef herd is 27. In dairy herds, AI is predominantly used (71% AI vs. 29% stock bull) while stock bulls are predominantly used in dairy‐beef and beef herds—66% stock bull versus 34% AI and 78% stock bull versus 22% AI, respectively.

The routine genotyping of all cattle at birth will play an important future role in the management of genetic diseases firstly by having a tissue sample for DNA testing collected at birth and secondly by having a record of DNA‐verified parentage. Other opportunities include the routine genotyping of major genes or traits of importance for reporting, management of inbreeding and lethal conditions such as lethal recessive haplotypes through mating support tools, and detection of chromosomal regions of homozygosity and aneuploidy.

In tandem with this, the routine recording of congenital defects by veterinary practitioners, pathologists, farmers and researchers is a collective approach that can be taken to identify abnormal phenotypes. While this occurs currently on an ad hoc basis internationally, more formal structured national recording programmes are required. This study presents results from a longitudinal survey of congenital defects reported to the Irish Cattle Breeding Federation (ICBF).

## 2 Materials and Methods

### 2.1 The Survey

The Irish Cattle Breeding Federation (ICBF) provides cattle breeding information services to the Irish beef and dairy industry (www.icbf.com). A survey was conducted by the ICBF over a 10‐year period (2014–2023) to survey the congenital defects occurring in the Irish national cattle population.

A questionnaire survey, consisting of 33 questions (Appendix S1) was designed and formatted using an on‐line platform (www.surveymonkey.com) and accessible via the ICBF website. The questions were a mix of both open‐ended and close‐ended questions. The first 10 questions collected epidemiological information about the individual reporting the defect, the status of the animal (dead/alive), level of dystocia (with/without difficulty), gestation length (premature/at term/over term) and whether it was a single or a twin. Questions 11 to 30 collected data on the type of defect being reported by body system; in the majority of cases, reports were filed by herdowners and hence did not include details of necropsy examinations. The final three questions aimed to prompt the respondent to send pictures and a hair sample to ICBF and to enter contact details in the event that they were willing to be contacted by ICBF for further information. Any herdowner, farm worker or veterinarian could participate; questions were not designed for particular respondent groups (e.g., farmers, vets). Respondents did not need to be a member of ICBF. Of total respondents, 74% were the affected animal owners. Of the 26% who were not, 72% of these were veterinarians, 5% were farm workers, 1% were breed societies and the remainder (22%) were unknown. The survey was promoted sporadically via web posts, social media and agricultural news platforms. Respondents were not incentivised to participate in the survey.

Each of the responses to questions 11 to 30 on the type/s of congenital defect reported were reviewed independently by both authors then a consensus was reached to categorise each case into one of 16 congenital defect categories. The descriptors used to assign cases to each category are shown in Table 1. An ‘other’ category was included to group together cases where fewer than or equal to five cases of an individual defect was recorded.

**TABLE 1:** Categories of congenital defects reported in this survey ranked in descending order of number of cases per category and category descriptors (*n* = 522 cattle).

Category	Cases (no.)^a^	Frequency (%)^a^	Descriptors of congenital defects in each category
Atresia	127	24.3	Unable to pass faeces, incomplete/no back passage, waterbelly, incomplete gut, blocked bowel, missing part of gut, enlarged belly/stomach/abdomen, fluid‐filled abdomen, segmented bowel, atresia jejuni, coli, ani.
Multiple	104	19.9	At least two defects in the same animal
Tail	70	13.4	Anury (*n* = 58), short tail (7), wry and short tail (2), deformed tail (2), wry tail (1)
Leg	38	7.3	Ankylosis (20), leg defect (11), hoof defect (4), contracted tendons (3)
Other	30	5.8	< 5 cases/individual defect category, e.g., ear defects (4), polydactly (3), amelia (4), amorphous globosus (2), …
Dwarf	21	4.0	Much smaller calf than normal, shorter than normal legs
Genito‐urinary	17	3.3	Genito‐urinary defect (7), testicular defect (3), cervical defect (1), ovarian defect (1), uterine defect (1)
Behavioural	17	3.3	Star gazing (5), nervous signs (3), spastic paresis (2), spastic syndrome (3), seizures/fits (1), uncoordinated (3).
Skin or hair	16	3.1	Skin defect (7), alopecia (6), hypotrichosis (3)
Cleft	15	2.9	Palate (7), muzzle (6), palate/muzzle (2)
Heart	15	2.9	Ventricular septal defect (9), atrial septal defect (3), heart defect (3)
Eye	14	2.7	Anophthalmia (6), blind (4), cataracts (2), eye defect (2)
Omphalocele	12	2.3	Enterocoele (5), viscera/organs out through navel (4), abomasocoele (3)
Spinal	11	2.1	Torticollis/wry neck (4), spina bifida (2), perosomus elumbis (2), brachyspina (1), kyphosis (1), vertebral defect (1)
Schistosomus reflexus	8	1.5	Inside out calf, all organs outside body and back twisted, calf folded over on itself
Hydrocephalus	7	1.3	Big head full of fluid, big domed, soft skull

^a^ Other cases may be recorded in the multiple defect category; whether a complete or partial necropsy by a veterinary practitioner or a veterinary laboratory pathologist was performed not recorded.

### 2.2 Epidemiological Data

Between 2014 and 2023 inclusive, 575 cattle with congenital defects were reported by respondents. Following the removal of duplicate reports (same case reported twice in a short time period suggesting respondent feared initial response had not been uploaded), reports relating to non‐congenital defects (reports of ill‐health in cattle but no details of a congenital defect reported) and reports where the defect could not be determined based on the information provided, 522 reports remained. Of the 522 unique reports, 369 cattle were officially registered (correct tag number) with the Department of Agriculture, Food and the Marine (DAFM). In Ireland, breeders are required to tag and register a calf within 28 days of birth, however, many aborted and stillborn calves may not be tagged contributing to the ‘untagged calf loss phenomenon’ (Mee 2020). Where a tag was officially registered, additional information could be retrieved from the ICBF database. These data (birth, breed and herd location details) were available for 369 animals born in 251 unique herds. Breed type included both purebred and cross breds as registered by the breeder. An animal's assigned breed type was based on the breed of the sire and dam. The extent of calving assistance provided at an animal's birth, together with date and type of breeding service, dam parity (after affected calf birth) and single/twin data were also available for some animals; farmers are not legally obliged to provide this information when registering a calf. Calving assistance is scored on a 1–4 scale where 1 = unassisted, 2 = slight assistance, 3 = considerable difficulty and 4 = veterinary assistance. Gestation length was calculated as the number of days between the last available service date and the subsequent calving date, provided the sire recorded for the resulting progeny was also the recorded service sire.

## 3 Results

### 3.1 Epidemiological Information

**FIGURE 1:**
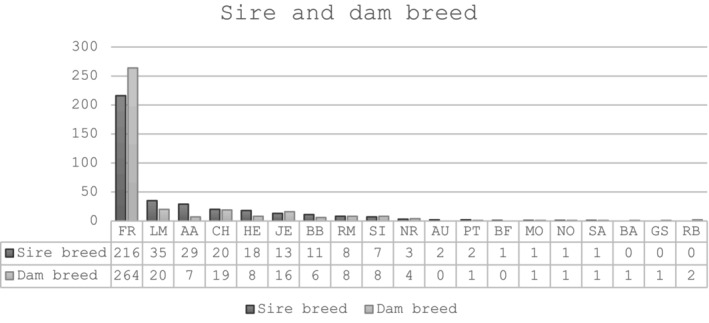
The sire and dam breed of calves registered with a congenital defect (*n* = 369). For details of breed codes see https://assets.gov.ie/90238/f5f58c25‐4cf6‐400f‐a2f0‐8b460758716a.pdf.

Birth, breed and herd location data were available for all 369 affected cattle (Table 2). Approximately 63% of cases reported came from a dairy herd and 36% from a beef herd. Of this number, 170 calves were registered as female and 199 calves were registered as male. The predominant sire breed was Friesian, which includes both Friesian and Holstein (FR), followed by Limousin (LM), Aberdeen Angus (AA), Charolais (CH), Hereford (HE), Jersey (JE) and Belgian Blue (BB) while the most common dam breeds were FR, LM, CH, JE, HE and Simmental (SI) (Figure 1). Of the sire breed × dam breed type categories (dairy × dairy, beef × beef and dairy × beef), there were 235, 80 and 54 affected animals, respectively (Table 1). A minority (*n* = 17) of calves were registered as twins.

**TABLE 2:** Epidemiological data for all ICBF‐registered calves (369) reported with a congenital defect and for each of the three most common defect categories of registered calves: atresia (*n* = 85), multiple defects (73) and tail defects (40).

Trait	Trait category	All defects	Atresia	Multiple defects	Tail defects
		369	85	73	40
Gender	Male	199	56	40	17
	Female	170	29	33	23
Breed type	Dairy × Dairy	235	54	47	29
	Dairy × Beef	54	18	11	9
	Beef × Beef	80	13	15	2
Single/twin	Twin	17	3	6	2
	Single	352	82	67	38
Service	AI	237	62	41	29
	Natural	11	4	0	3
	Unknown	121	19	32	8
Parity	1	50	7	12	3
	2	61	14	10	2
	3	62	17	12	3
	4	51	12	12	9
	5	53	17	6	7
	6	36	8	7	5
	7	20	2	7	6
	8	19	6	3	3
	9	7	1	2	1
	10	1	1	1	1
	11	4	0	1	0
	Unknown	5	0	0	0
Calving ease	1	243	60	40	34
	2	45	10	10	2
	3	21	9	3	1
	4	18	1	8	0
	Unknown	42	5	12	3

Of the four provinces, the majority of reports (232) were for herds in Munster, with 92, 27 and 17 reports for herds in Leinster, Connacht and Ulster, respectively. Thus, 63%, 25%, 8% and 4% of cases came from Munster, Leinster, Connacht and Ulster, respectively; the equivalent data for the provincial distribution of the national cattle population are 54%, 28%, 11% and 7%, respectively.

Data regarding dam parity were available for 364 animals. The majority of dams were in their 3rd or higher (*n* = 253) lactation when giving birth to the calf with the reported congenital defect (Table 2). Calving ease data were available for 327 dams, of which 243 calved without assistance, 45 required some assistance, 21 required considerable assistance and 18 calved with veterinary assistance. Breeding data were available for 248 calves, of which 237 were sired by AI bulls and 11 were sired by natural service sires (Table 1). Gestation length ranged from 252 to 299 days.

### 3.2 Congenital Defect Information

Congenital defect records for the 522 animals were categorised as shown in Table 1. The three most commonly recorded defect categories were intestinal atresia alone (24.3%), multiple defects (19.9%) and tail defects alone (13.4%); these accounted for over half of all congenital defects recorded (57.6%).

#### 3.2.1 Intestinal Atresia

**FIGURE 2:**
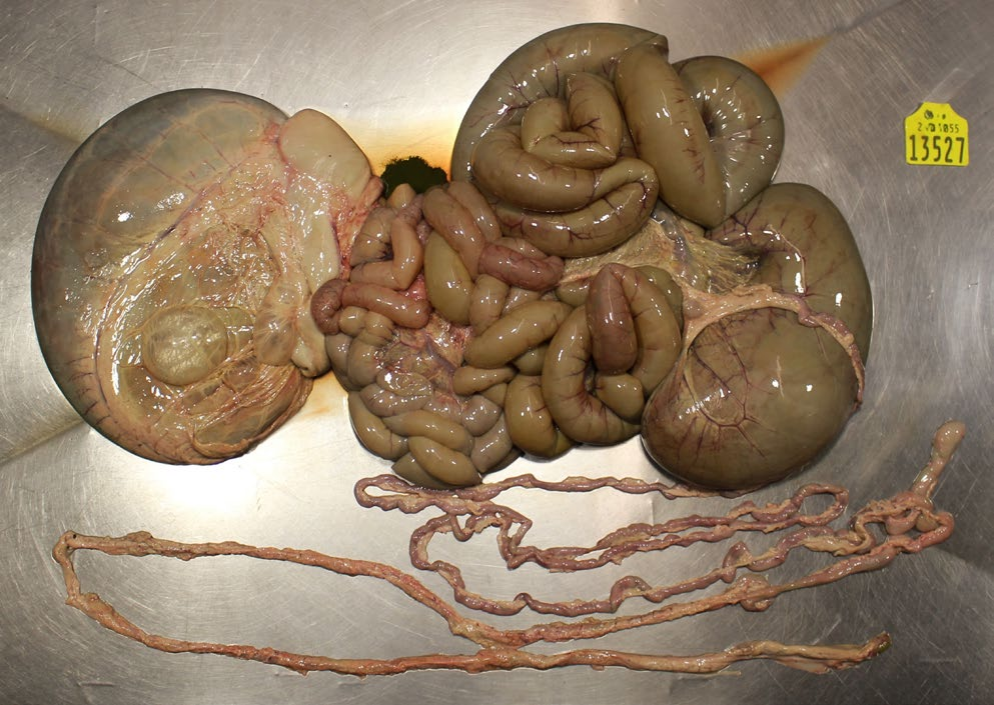
Intestinal atresia was the most commonly reported congenital defect.

Of the 127 atresia cases recorded (Figure 2), 85 were registered with ICBF; hence additional data were available (Table 2). The majority (56) of these calves were male, and the predominant breed type was dairy × dairy (54). The majority of calves were born unassisted (60). The majority of dams of these calves were served by AI (62) and most dams of affected calves were pluriparous (92%). Note, in addition to the 127 calves with atresia alone, there were an additional 20 calves with atresia reported alongside another defect; these are reported in the multiple defect category below—a total of 147 calves with atresia (28.2% of all congenital defect cases).

#### 3.2.2 Multiple Defects

**FIGURE 3:**
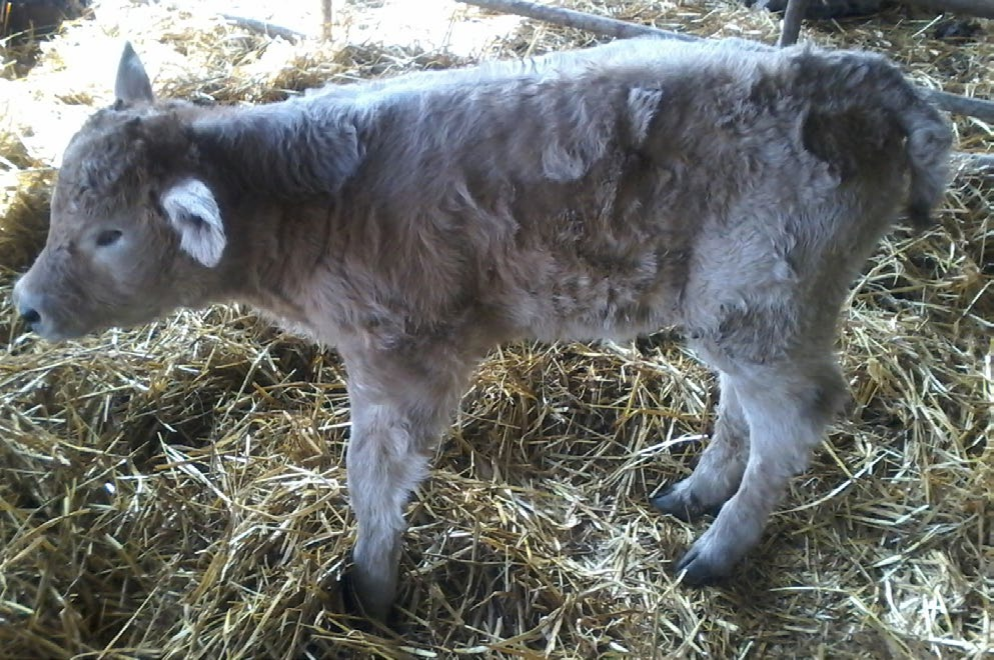
The second most commonly reported congenital defect category was multiple defects.

The second most common defect category reported was multiple defects (Table 1), (Figure 3). Further data were available on 73 of these registered cases. The majority of these calves were male (40), born unassisted (40) and were dairy × dairy (47) and AI‐bred (41). The majority of dams of affected calves were pluriparous (84%).

The 10 most common defect categories (each case of which had a co‐defect) within the multiple defect category are shown in Table 3. The three most common of these defect categories, within the multiple defect category, were tail defects (27.5% of all multiple defect cases), ankyloses (19.6%) and intestinal atresia (19.6%). Each of these defect categories had 18, 16 and 18 different co‐defects, respectively, that is, a case with a tail defect could have any of 18 different individual or multiple co‐defects. Including calves with a single defect and those with multiple defects, 28/98 tail defect calves (28.6%) and 20/147 (13.6%) atresia calves had a co‐defect. The co‐defects most frequently associated with each of the three most common defect categories were: tail defect and eye defect (of 28 tail defect cases with a co‐defect, 13 also had an eye defect; 46.4%), ankyloses and scoliosis (5/20; 25%) and intestinal atresia and a tail defect (6/20; 30%).

**TABLE 3:** Ten most common co‐defects in the multiple congenital defects category recorded in this survey, ranked in descending order: number of cases per category.

Category	Cases (no.)
Tail^a^	28
Ankylosis	20
Intestinal atresia	20
Eye	19
Kyphosis	18
Clefts	13
Dwarf	12
Hydrocephalus	9
Brachygnathia	9
Omphalocoele	7

^a^ Each of these were recorded with a co‐defect, for example, tail defect with an eye defect.

#### 3.2.3 Tail Defects

**FIGURE 4:**
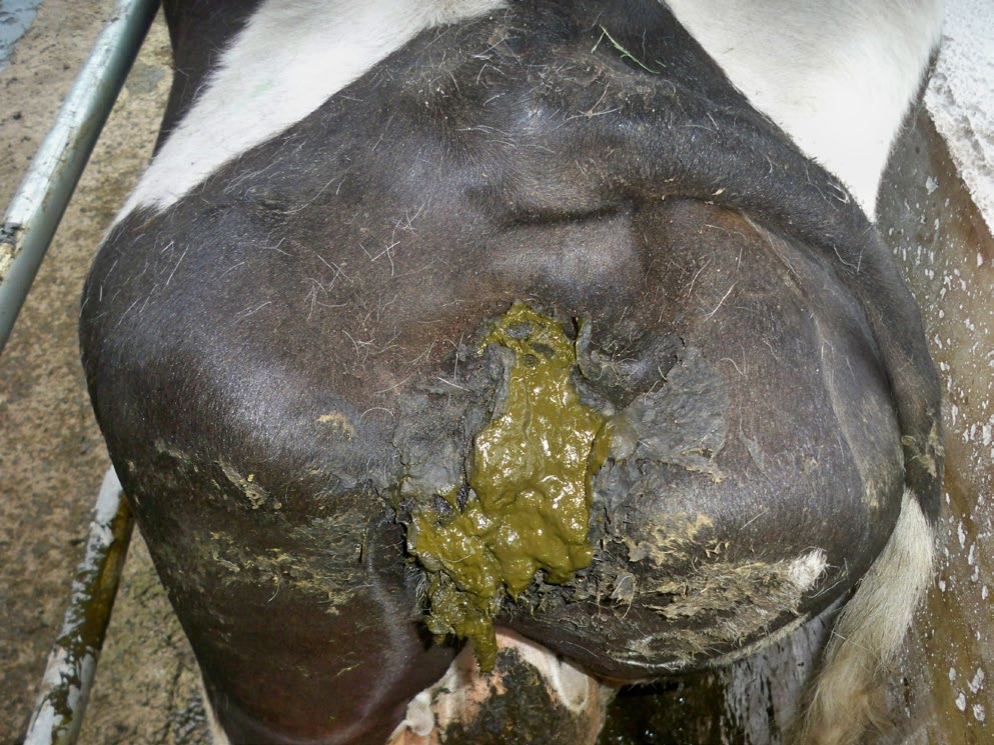
The third most commonly reported category of defects was tail defects.

The third most commonly reported category of defects was tail defects, accounting for 13.4% of cases (Table 2), (Figure 4). Further data were available on 40 registered calves of the 70 cases. The majority of calves were female (23) and the result of dairy × dairy mating (29), and of an AI service (29) and were born unassisted (34). The majority of dams were pluriparous (93%). Note, in addition to the 70 calves with a tail defect alone, there was an additional 28 calves with a tail defect reported alongside another defect; these are reported in the multiple defect category above—a total of 98 calves with a tail defect (18.8% of all congenital defect cases).

Similar to these three defect categories (atresia, multiple, tail defects), the majority, though not all, of the other defects reported were externally visible and not requiring a necropsy to diagnose their presence, for example, dwarfism, schistosoma reflexus and amorphous globosus.

## 4 Discussion

This study demonstrates a model of congenital defect recording, with use of accessory data from a national breeding organisation, which may be replicated internationally. Hence, this is a study of international relevance. The external validity of the study can be established by comparing the key findings reported with other congenital defect studies both nationally and internationally. For example, the finding that atresia was the most commonly reported defect aligns well with previous Irish studies; submissions of cattle with congenital defects by farmers to the Irish veterinary laboratories service (passive surveillance), (Johnson 2022), an Irish dairy whole‐herd calf mortality study (active surveillance), (Mee 2015) and a recent survey of bovine congenital defects conducted by Irish private veterinary practitioners (Mee et al. 2024).

This is the first national survey of bovine congenital defects in Ireland. It is also one of the largest, if not the largest, studies documenting two of the most common cattle defects, atresia and tail defects, in the world. This is the power of this model of herdowner recording supported by a national database containing demographic information on affected animals. It can detect unique signals in the data usually not recorded in veterinary case reports of congenital defects, for example, the parity profile of dams of atretic or tail defect calves.

Uniquely, this study of farmer‐reported congenital defects gives the best perspective of the true typology and epidemiology of congenital defects on‐farm. Reporting by all other sources, for example, private veterinary practitioners, veterinary pathologists and veterinary researchers represents a biased sample of these defects. For example, farmers in this study reported both live and dead and healthy and unhealthy cattle with defects. However, PVPs tend to only attend unhealthy cattle, pathologists only see dead cattle and researchers tend to only observe selected live or dead, healthy/unhealthy cases. Given these divergent perspectives, it is not surprising that the results from studies conducted with different groups of respondents/observers will yield divergent findings on congenital defect typology and epidemiology. This is clear from the high relative prevalence of tail defects in this study (almost 20% of all cattle with congenital defects)—these are not reported at all in studies conducted on congenital defects from pathology laboratories (e.g., Johnson 2022).

The finding that atresia was the most commonly reported defect by Irish farmers is somewhat surprising in an international context. The most commonly diagnosed bovine congenital defect reported internationally has variously been attributed to umbilical hernia (e.g., Polat 2022) or arthrogryposis (e.g., Bähr and Distl 2005). However, atresia has consistently been reported as a frequent defect in Irish cattle at animal‐ and herd‐levels (Mee and Kenneally 2010; Mee 1994) following first documentation in the 1960s (McGeady et al. 1967). This may suggest an under‐reporting internationally due to the submission biases inherent in many bovine congenital defect studies or an underlying international difference in the risk factors for, and hence prevalence of, atresia.

The finding in the present study that the majority of calves with atresia were male (66%) is consistent with previous Irish (Mee et al. 2024; Keane et al. 2023) and international studies (Zilci and Kılıç 2019). Similarly, the finding that the majority of dams of atretic calves were pluriparae (71%) is consistent with previous Irish reports (Mee et al. 2024; Keane et al. 2023). Very few international studies report the parity of dams of affected calves; Nihleen and Eriksson (1958) reported that six of the seven dams of affected calves were pluriparous. Of atresia cases reported in the present study, 3.5% were reported in twin calves. Atresia has occasionally been reported in one of the twin pair internationally, varying between 1.5% and 7.6% of all atretic calves (Rademacher et al. 2002; Smith et al. 1991). In a minority (13.6%) of atretic calves, a co‐defect was reported, most commonly a tail defect. The most commonly reported co‐defects with atresia have been limb, kidney and tail defects (Smith et al. 1991; Ducharme et al. 1988; Azizi et al. 2010). Unfortunately, in the present study, it was not possible to differentiate between the various types of atresia (e.g., atresia jejunum, ileum coli, ani) to see whether particular types were more likely to co‐occur with certain other defects.

The cause of congenital intestinal atresia has been investigated previously, and an underlying genetic basis has been hypothesized based on pedigree analysis (Čítek et al. 2022; Syed and Shanks 1992; Nihleen and Eriksson 1958), a candidate gene or causal mutation has not been identified to date. Keane et al. (2023) suggested that atresia may be a complex multigenic condition rather than a simple Mendelian trait involving a single locus following a GWAS carried out on dairy calves identified with intestinal atresia in Ireland. The results of that study found three related Jersey sires produced progeny with a higher incidence of intestinal atresia; however, no clear candidate genes were identified. To further support the complexity of this trait, atresia can occur in the small and large intestine, from the duodenum to the anus, and whether atresia in each location has different underlying causes has yet to be established (Mee et al. 2024).

The second most commonly reported congenital defect category was multiple defects. The proportion of affected calves reported (20%) probably underestimates the prevalence of congenital defect co‐occurrence as there may have been other internal co‐defects in other calves which were not observable externally and so went unreported. Additionally, some calves with a single defect recorded may have had other, unrecorded defects, also contributing to the underestimation of the prevalence of multiple defects. Reports on the prevalence of multiple defects in population studies are scarce and highly variable. In a recent Irish study, 13.1% of deformed calves had multiple defects (Mee et al. 2024), in Turkish studies the proportion of deformed calves with multiple defects varied between 0.9% and 15.7% (Sağlam et al. 2022; Karasu et al. 2024) while in a Finnish study, 72% of deformed calves had multiple defects (Oksanen 1972). Within the multiple defect category in the present study, the top five reported defects were tail defects (reported with a co‐defect), ankylosis, atresia, eye defects and kyphosis. The musculoskeletal system, in particular ankylosis, has previously been reported as the most common body system involved in multiple defects (Mee et al. 2024). Congenital defects that co‐exist may arise as a result of spontaneous de novo mutations or as part of a syndrome emanating from a single cause (e.g., crooked calf syndrome—Welch et al. 2024). There was some evidence from the present study that particular defects were more likely to co‐occur, for example, tail and eye defects, suggesting a syndromic occurrence. This link has been observed previously in multiple cattle breeds (e.g., Reddy et al. 2018). However, the multitude of combinations of different defects co‐occurring in most cases in the present study indicated that most multiple defect cases were probably the result of unrelated multiple de novo mutations involving multiple genes (Marc et al. 2023). Tail defects were the third most common category (13%) of which the majority were anury or brachury. While there are multiple case reports of congenital tail disorders in calves (e.g., Lotfi and Shahyryar 2009) there is a paucity of case series data and the prevalence data are highly variable. For example, tail defects were recorded in 28% of deformed calves in various German cattle breeds (Szabo 1989), in between 0.7% and 8.9% of Turkish deformed calves (Polat 2022; Belge et al. 2000; Sağlam et al. 2022) and in 1.6% of Irish deformed calves attended by a veterinary practitioner (Mee et al. 2024). As tail defects are non‐lethal and affected calves are generally unaffected by the condition (apart from being unable to swat away flies), many such cases go unreported to veterinary practitioners or veterinary laboratories so apparent prevalence data are likely to grossly underestimate the true prevalence of these conditions.

In a study of taillessness in a cloned cow, the authors concluded that taillessness does not have a genetic origin; rather, it occurs as a result of an epigenetic programming error during development in the normal cattle population (Wagner et al. 2017). Thus, cows that produce a calf with no tail are highly unlikely to do so again, and tailless heifers are also unlikely to produce tailless calves.

Of the other defects reported, the majority were visible externally, a common feature of farmer‐ and PVP‐reported defects. Thus, by comparison with veterinary laboratory (necropsy)‐reported defects (e.g., Johnson 2022; 39%), the proportion of calves with, for example, cardiac defects reported here (2.7% of cattle with congenital defects) was much lower. This illustrates how the source of the recorded defects biases the typology of the defects recorded.

This study had some limitations. Firstly, much of the information recorded in the survey was self‐reported by farmers and unverifiable as in most cases a veterinary clinical or necropsy examination was not conducted. This limits our ability to determine whether, for example, intestinal atresia was more common than atresia ani, which may be relevant for potential genetic control measures. Similarly, there was a bias towards externally visible defects in the study, with very few internal defects recorded.

There were also some advantages to this study design. While a high‐quality professional diagnosis cannot be provided by a herd owner, they are uniquely positioned to report issues first‐hand. This ensures that the congenital defects reported are unaffected by selection bias, that is, cases are reported whether a veterinary practitioner was called to attend the calf's birth or euthanise the calf or not. This allows economically less important defects to be recorded, for example, many tail defects.

Overall, the results of this survey provided accurate findings in line with other studies and suggest that targeting herd owners as a means to surveil congenital defects may be an effective approach to monitor the probable true types and frequencies of defects nationally. Following this analysis and based on feedback, the survey has been reformed to make the survey more accessible, concise and convenient for all participants.

## Author Contributions

K.Q. managed the database, extracted the epidemiological data, and wrote the first draft of the paper. J.F.M. conceptualised the study, assigned the congenital defects to categories, redrafted the paper, and co‐wrote and submitted the final draft of the paper.

## Conflicts of Interest

The authors declare no conflicts of interest.

## Supporting information


Appendix S1.

## Data Availability

The data generated during the current study are available from K.Q. upon reasonable request.
