# Supplementary material for: Bovine Congenital Defects Recorded in a National Survey of Dairy and Beef Herds Over Ten Years (2014–2023)
Source: Reprod Domest Anim. 2025 Apr 30;60(5):e70067. doi: 10.1111/rda.70067 (PMC12042788; doi:10.1111/rda.70067)
Supplement: Supplementary file 1 — Appendix S1. [file RDA-60-e70067-s001.docx]

**Genetic defect reporting survey**

**1. What is the status of the animal?**

Alive

Dead

*** 2. Are you the animal's owner?**

Yes

No

If no, what is your role with the calf (Veterinarian, farm worker, knackery, etc)?

*** 3. What is your herd number?**

*** 4. What is the dam's tag number?**

**5. What is the sire's tag or ID number? (If unknown leave blank)**

*** 6. What is the animal's tag number?**

I am not tagging this animal

Calf tag number

**7. If the animal is a calf, was it aborted?**

No

Yes, premature (Approximately < 200 days)

Yes, premature (Approximately 200-240 days)

Yes, premature (Approximately 240-260 days)

Yes, at term (260-270 days)

Yes, over term (>270 days)

**8. How was the animal calved?**

Aborted

Without help

With help

Difficult

Caesarean section

*** 9. Is the animal alive?**

Yes

No

If no, how long after birth did it live?

* **10. Was the animal a single birth?**

Yes

No, it was a twin

No, it was a triplet

No, it was conjoined

Other (please specify)

* **11. Where do you see abnormalities?**

Skin, Hair coat

Whole body

Head and Neck

Spine, Shoulders, Hips, Limbs

Abdomen, Internal organs, Genitalia, Anus

Behavior

Specific diagnosis

*** 12. Are you planning on sending the animal for a postmortem examination?**

Yes

No

If yes, which laboratory

**13. Is there an abnormality with the skin?**

Scaly or thickened

Missing patches of skin

Missing color

Tumor

Other/Specific diagnosis

**14. Is there an abnormality with the hair coat?**

Albino (White hair, pink skin and pink/red eyes)

Light hair coat

No hair

Other/Specific diagnosis

**15. Are there any developmental abnormalities?**

Mummified

Weak calf

Small hairy mass without legs or obvious head

Much smaller than normal (Dwarf)

Much larger than normal

More muscular than normal

Less muscular than normal

Other/Specific diagnosis

**16. Is there an abnormality with the head?**

Too big

Too small

Fine/elongated

Dome shaped

Distorted/twisted

Other/Specific diagnosis

**17. Is there an abnormality of the skull?**

Soft skull

Hole in skull possibly covered by skin or hair

Other/Specific diagnosis

**18. Is there an abnormality of the eyes?**

Abnormal shape

Abnormal placement

Missing

White Spots in the eye

Twitching eyes

Bulging eyes

Night vision problems

Progressive loss of vision

Other/Specific diagnosis

**19. Is there an abnormality of the mouth?**

Abnormal opening of the mouth

Split upper lip

Split lower lip

Tongue too large

Tongue too small

Ulcers in the mouth

Loss of teeth

Excessive salivation

Difficulty eating

Difficulty drinking

Lower jaw protrudes farther than the upper jaw

Upper jaw protrudes farther than the lower jaw

Split nostrils

Partial or total blockage of the nostrils

Other/Specific diagnosis

**20. Is there an abnormality of the neck?**

Too thick

Too short

Too long

Twisted

Absent

Two heads

Other/Specific diagnosis

**21. Is there an abnormality of the spine?**

Hunchbacked

The middle of the back dips below the shoulders and the hips

Twisted to the side

Open

Folded over on itself (schistosome)

Deformed tail

Missing tail

Other/Specific diagnosis

**22. Is there an abnormality of the shoulders?**

Too narrow

Too wide

Other/Specific diagnosis

**23. Is there an abnormality of the hips?**

Too narrow

Too wide

Other/Specific diagnosis

**24. Is there an abnormality of the legs?**

Missing one leg

Missing two legs

Missing three legs

Missing all legs

Extra legs

Shorter than normal legs

Longer than normal legs

Twisted/rotated

Contracted tendons

Fixed joints

Please specify which leg(s) are affected

**25. Are there abnormalities of the hooves?**

Missing part of the hoof or claw

Extra hoof

Joined hooves-mule foot

No hoof

Scissor hoof

Corkscrew claw

Plesae specify on which leg the deformity occurs

**26. Is there an abnormality of the abdomen or internal organs?**

Swollen, full of fluid

Visible viscera/organs through the navel

Navel hernia (no organs visible)

Lung abnormality

Liver abnormality

Heart defect/abnormality

Intestine abnormality

Other/Specific diagnosis

**27. Is there an abnormality of the genitalia?**

Absent

Incorrectly positioned on the body

Deformed

Hermaphrodite, both sets of genitalia present

More than 2 testicles

Less than 2 testicles

Free martin

Other/Specific diagnosis

**28. Is there an abnormality of the anus**

Absent

Prolapsed

Thick mucus at anus/not first dung

Other/Specific diagnosis

**29. If alive, does the calf behave abnormally?**

Tremors/seizures

Turns in a circle

Always turns in the same direction

Holds head vertical in the air

Immobility/paralyzed

Lethargic

Uncoordinated

Unable to stand without assistance

Other/Specific diagnosis

**30. How long after birth did the calf begin displaying behavioral abnormalities?**

At birth

Hours after birth

Within the first week after birth

Within the first month of birth

Within the first 6 months of birth

Within the first year of life

Within the first 2 years of life

*** 31. Would you be willing to take pictures of the animal and e-mail them to us ? If yes, e-mail them to Health@ICBF.com**

Yes

No

*** 32. Would you be willing to send us a tissue or hair sample for DNA analysis? If Yes, you may be issued a collection kit from ICBF.**

Yes

No

*** 33. May we contact you if we have any questions about this animal in the future? (If Yes, Please provide your name, and a phone number or e-mail address where we can contact you in the box below)**

Yes

No

Please provide a name and phone number or e-mail address where we can contact you
